# A 7-Year Retrospective Multisource Analysis on the Incidence of Anesthesia Awareness With Recall in Cancer Patients: A Chance of Collaboration Between Anesthesiologists and Psycho-Oncologists for Awareness Detection

**DOI:** 10.1097/MD.0000000000002757

**Published:** 2016-02-08

**Authors:** Marco Cascella, Daniela Viscardi, Vincenzo Schiavone, Farrokh Mehrabmi-Kermani, Maria Rosaria Muzio, Cira Antonietta Forte, Francesco De Falco, Daniela Barberio, Arturo Cuomo

**Affiliations:** From the Division of Anesthesia, Department of Anesthesia, Endoscopy and Cardiology Istituto Nazionale Tumori “Fondazione G. Pascale”— IRCCS, Naples, Italy (MC, DV, AC); Division of Anesthesia and Intensive Care, Hospital “Pineta Grande,” Castel Volturno, Caserta, Italy (VS); Division of Neurosurgery, Hospital “Pineta Grande,” Castel Volturno, Caserta, Italy (FM-K); Division of Infantile Neuropsychiatry, UOMI—Maternal and Infant Health, Asl NA 3 SUD, Torre del Greco, Naples, Italy (MRM); Psychology, Division of Pain Medicine, Department of Anesthesia, Endoscopy and Cardiology Istituto Nazionale Tumori “Fondazione G. Pascale”— IRCCS, Naples, Italy; Psychooncology, Department of Quality of Life, Istituto Nazionale Tumori “Fondazione G. Pascale”—IRCCS, Naples, Italy (FDF, DB).

## Abstract

Although randomized controlled studies reported an incidence of anesthesia awareness with recall ∼1 to 2 per 1000 (0.1–0.2%), recent data from the NAP5 study showed an incidence of only 1:19,600. Although in a prospective study many tools for anesthesia awareness detection can be used, a retrospective analysis requires a careful collection of information.

The aim of the study was to evaluate the incidence of anesthesia awareness with recall in a cohort of cancer patients through a multisource retrospective analysis, and the clinical description, including the psychological outcome, of the cases detected. We also tested whether our retrospective analysis would be improved by a routinely psycho-oncological assessment. As secondary endpoints we evaluated the use of depth of anesthesia monitoring over a large cohort of patients, and the correlation between the brain monitoring and the incidence of awareness.

We have carried out a 7-year retrospective analysis in a large cohort of cancer patients on the incidence of awareness with recall during general anesthesia. Of 35,595 patients assessed for eligibility, 21,099 were studied. We analyzed all data from the operative rooms’ database, the anesthesia records, and from the database of the surgical divisions. In addition we examined reports from psychologists and spontaneous reports to the quality team of the hospital.

Two certain cases of awareness were detected, with an incidence of 1:10,550 (0.0095%). They occurred during elective surgery, in female patients without other risk factors. One case came from the report of a psychologist. In both episodes, brain monitoring was not applied and no long-term psychological sequelae were reported.

Despite the limitations, our investigation suggests that the incidence of anesthesia awareness is very low, also in a specific cohort of patients, such as the cancer patients, and even when the depth of anesthesia monitoring is rarely used. The limitations caused by both the retrospective analysis and the absence of specific tools for direct awareness detection, such as structured interviews, can be filled with an effective postoperative psychological assessment which is often of routine in a cancer center. This observation could suggest the usefulness of inserting specific questions within the psychological tools commonly used by psycho-oncologists.

## INTRODUCTION

Historically, the phenomenon of general anesthesia awareness with recall (GAAWR) is one of the greatest fears in anesthesiology. This anesthesia complication is the explicit recall of sensory perceptions of the patient during anesthesia;^1–3^ thus, it represents a paradox as the purposes of the anesthesia are both unconsciousness and amnesia. Although GAAWR is a well-described phenomenon, the literature offers contradictory data on its real incidence. Several randomized controlled studies reported an incidence of GAAWR ∼1 to 2 per 1000 operations involving general anesthesia (0.1–0.2%), in the absence of risk factors and either with intravenous^4^ or volatile anesthetics, even if a Japanese report showed a higher incidence of awareness in total intravenous anesthesia.^5^ The data on the incidence of GAAWR regard reports scheduled in Western countries,^6,7^ whereas incidence in China is ∼0.41%.^8^ However, recent data from the 5th National Audit Project from Great Britain (NAP5), evaluated in >2.7 million cases, reported an incidence of awareness of only 1:19,600, that is 20 times less than previously reported.^9^ Some authors have criticized the results because of the methodology of data collection, especially in regard to the absence of structured interviews that may have underestimated the real incidence of intraoperative awareness.^10^ Another controversy concerns the use of depth of anesthesia (DOA) monitoring. Nowadays, we do not know the advantage in using DOA monitoring,^11^ because many conditions, such as age, race, gender, low core body temperature, acid-base imbalances, low blood glucose, drugs administered to the patient (eg neuromuscular blocking agents) or brain ischemia have a significant effect on the reliability of the most common used DOA devices. Additionally, DOA monitors are limited by their calibration range and the interpatient variability in their dose–response curves. Moreover, despite the refinement of the algorithms, it is impossible to clear the weight of all artifacts. In consequence, there is a large variability in the use of DOA devices during the anesthetic practice. According to the American Society of Anesthesiology (ASA), GAAWR is prevented by preoperative identification of patients at risk for GAAWR. In these patients there would be an advantage in brain-function monitoring, but only when used in association with clinical and standard instrumental monitoring of the anesthesia.^12^ In addition, a recent Cochrane review^13^ concluded that BIS-guided anesthesia and ETAG-guided anesthesia may be equivalent in protection against intraoperative awareness; however this assumption still requires certain evidences.

The objective of the study was to evaluate the incidence of GAAWR in a cohort of cancer patients through a multisource retrospective analysis, and the clinical description, including the psychological outcome, of the detected cases. Assuming the limitations of a retrospective analysis in studying the incidence of GAAWR, we discuss on the effectiveness and the possibility of inserting specific items within the psychological tools used by psycho-oncologists, for the purpose of detecting awareness. The secondary endpoint was to evaluate the use of DOA monitoring over a large cohort of patients, and the correlation between the brain monitoring and the incidence of GAAWR.

## MATERIALS AND METHODS

### Type and Setting of the Study

**FIGURE 1:**
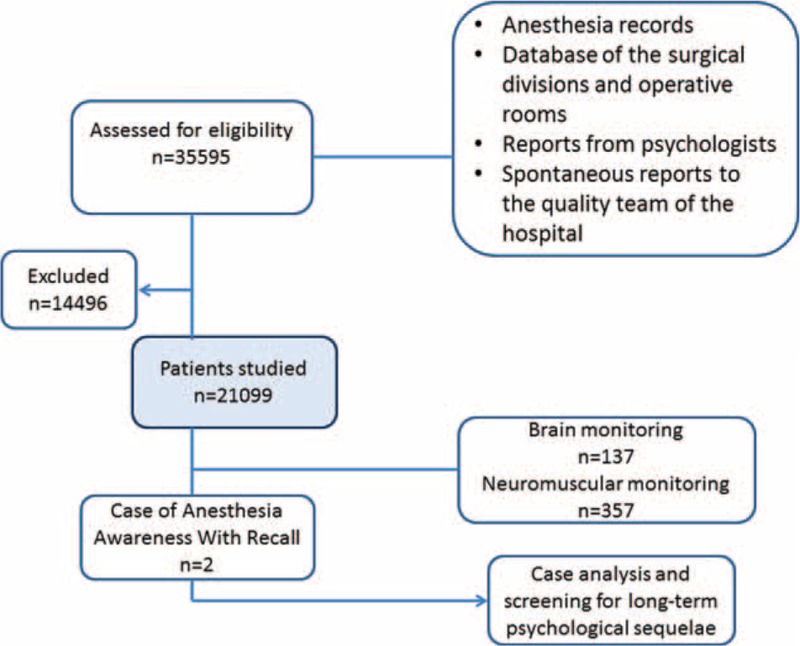
Flow diagram of the study.

A single center retrospective study on cancer patients was carried out from January 2007 to December 2013 at the Istituto Nazionale Tumori—Fondazione “G. Pascale,” Naples, Italy. The flow diagram of the study is reported in Figure 1. We analyzed all data from the operative rooms’ database, the anesthesia records, and from the database of the surgical divisions. In addition, we examined reports from psychologists and spontaneous reports from the patients to the quality team of the hospital. Because this study is a retrospective analysis, ethical approval was not necessary. Moreover, an informed consent module was developed for the description of any cases found.

The whole cohort included all the patients that underwent elective and nonelective surgery for cancer diseases, performed under general anesthesia or deep sedation. We evaluated the anesthesia features (inhaled, total intravenous, or combined regional-general anesthesia), the kind of surgery, the operative conditions (elective and nonelective surgery), the DOA monitoring, and the neuromuscular block monitoring data.

The patients’ experience was classified using the 5 class Michigan Awareness Classification (MiAC), Thus, class 1 is defined as isolated auditory perceptions, class 2 is tactile perceptions, class 3 is pain, class 4 is paralysis, and class 5 is paralysis and pain. According to this tool, an additional letter—“D” for distress—was also included in the presence of reports of fear, anxiety, suffocation, sense of doom, sense of impending death, and so on.^14^

We cleaned data excluding incoherent data (eg between manager reports and anesthesia reports), incomplete reports, operative reports with anesthesia or surgical division reports missing, absence or incomplete psychological assessment.

## RESULTS

Out of 35,595 patients assessed for eligibility, 21,099 (59.3%) were studied. Table 1 specifies the characteristics of the patients and surgery; the GAAWR cases, the anesthesia techniques, and brain and neuromuscular monitoring are shown in Table 2. The use of DOA monitoring was 137/21,099 (0.65%), whereas neuromuscular block monitoring was applied in only 357 studied cases (1.7%).

**TABLE 1:**
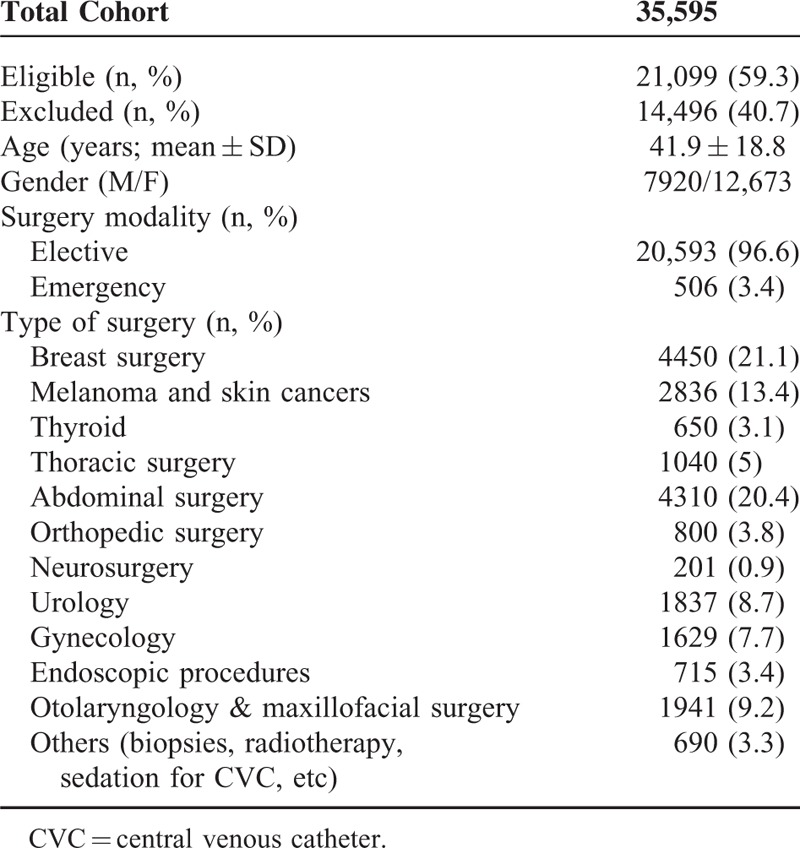
Characteristics of the Patients and Surgery

**TABLE 2:**
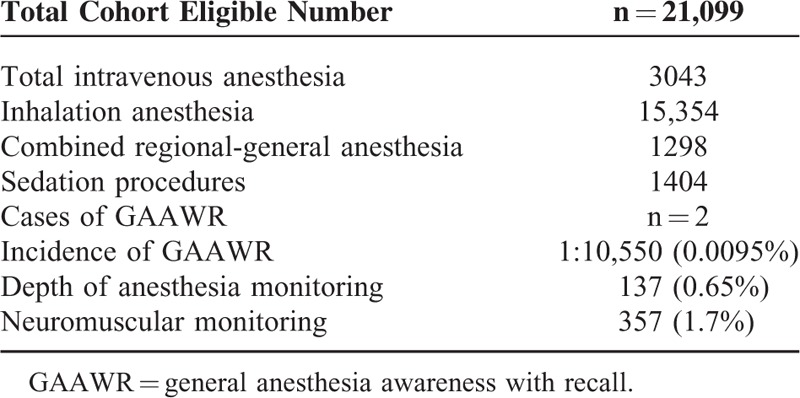
The Anesthesia Awareness Cases, the Anesthesia Techniques, and Brain and Neuromuscular Monitoring

From the available resources analyzed, we detected 2 certain cases of GAAWR corresponding to an incidence of 1:10,550 (0.0095%). Both patients were traced and have signed the informed consent to describe their clinical cases.

*Case 1*. A 37-year-old Caucasian woman of ∼65 kg, affected by breast cancer and scheduled for simple mastectomy with lymph nodes removal under the armpit, was presented. The anamnestic investigation showed that the patient had no risk factors for GAAWR, and she had never been operated previously. Before the beginning of the surgery midazolam 2 mg was administered as premedication. Anesthesia was performed with propofol 120 mg/kg and fentanyl 100 mcg administered at the induction, and fentanyl 100 mcg together with sevoflurane (ETAG > 0.7 MAC age adjusted) for the maintenance. According to the anesthesia record the concentration of sevoflurane was stable for all surgery time. Neuromuscular blockage was obtained with cisatracurium 12 mg. The brain monitoring and the monitoring of neuromuscular blockage was not used. The operative course was uneventful and the total operation time was 1 hour and 50 minutes. The recovery of consciousness at the emergence was sudden. Nevertheless, after a few hours the patient was spontaneously able to exactly report to a psychologist the specific dialog between the operator and a nurse in the operative theatre (MiAC Class 1). Most probably this conversation took place during the maintenance phase of anesthesia. This experience did not cause any distress, and the patient had no psychiatric sequelae in acute and long term.

*Case 2.* A 25-year-old Caucasian woman of ∼52 kg, who had not underwent any surgery before. Preoperative anamnestic investigation revealed that there were no risk factors for any postoperative complications. The patient was scheduled for total thyroidectomy for papillary thyroid carcinoma. After the premedication with midazolam 1.5 mg and fentanyl 100 mcg in the preoperative room, she received general anesthesia induced with propofol 110 mg, fentanyl 100 mcg and atracurium 25 mg, easily with facemask, ventilation and tracheal intubation. The maintenance of general anesthesia was performed with sevoflurane 2.5 % and fentanyl 200 mcg as total dose. Intraoperative monitoring for DOA and neuromuscular block were not applied. The total operation time was 2 hours and 45 minutes. Although there were no surgery complications during the intraoperative time, the anesthesia management became difficult because of continuous tachycardia and lachrymation of the patient. The possible cause was an erroneous administration of inhaled anaesthetics, and during the case analysis the anesthesiologist confirmed that the vaporizer was not well fixed to its support. He did not use inhaled anesthetic monitoring; therefore, the sudden arousal from the anesthesia status was not promptly detected. He also admitted that after about 5 minutes since he failed, he administered propofol to deepen the anesthesia status. There were no problems and patient complications at the emergence, but the day after surgery the patient spontaneously reported to the surgeon the experience of intraoperative pain without tactile or auditory perceptions. Nevertheless, she perceived a sense of suffocation and paralysis (MiAC Class 5 D). The psychological distress was resolved with minimal professional intervention, consisting in a few sessions of conversational psycho-diagnostic interviews, and fortunately she had no additional psychological complications.

The use of DOA monitoring was 137/21,099 (0.65%), whereas in only 357 studied cases (1.7%) neuromuscular block monitoring was applied.

## DISCUSSION

Is a retrospective analysis effective for detection of GAAWR^15^? As stated by Mashour et al^16^ there are strong methodological implications which reduce the resolution of a retrospective database analysis (see the section *Limitations of the study*). However, in our data analysis, 1 of the 2 cases of GAAWR came from a specific source, that is the report of a psychologist. Accordingly, in this study rather than to affirm the accuracy of a retrospective analysis for assessing the incidence of GAAWR, we use this finding to highlight the observation that the possibility of working in a team in which a psychologist is present could improve the power of detecting this complication. Unfortunately, this cannot be always possible; however, in a cancer hospital we can avail ourselves of the collaboration of psycho-oncologists, even to study perioperative problems such as awareness.

This study, moreover, offers the occasion to describe 2 interesting reports of GAAWR. The analysis of case 1 did not show any apparent cause for GAAWR. This finding is not surprising because, according to Bergaman's root case analysis, in 16% of cases, the awareness incident has no obvious cause.^17^ Thus, we investigated on the possible risk factors. As suggested by many authors, certain patient characteristics may be associated to an increased risk of GAAWR, including age, ASA physical status, and drug resistance or tolerance.^18^ Moreover, it has been reported an increased incidence of GAAWR in women (3 times more frequent)^19^ and in patients who underwent certain procedures, such as cesarean delivery,^20^ cardiac surgery,^21^ trauma surgery, rapid sequence induction anesthesia,^22^ and when the anesthesia management was performed without neuromuscular block monitoring,^23^ which should be applied even when using short-acting neuromuscular blocking agents. In our case we identified 2 risk factors: the female gender—maybe responsible for a resistance or tolerance to anesthetics—and the lack of neuromuscular monitoring. However, the missed use of neuromuscular monitoring is often responsible for awareness during emergence from anesthesia, whereas in our case the recall concerned the phase of maintenance.

The cause of the episode of GAAWR in case 2 was an error in the inhaled anesthetics administration. This report should not astonish because recent incident reporting studies suggest a rate of drug administration error ∼7/1000 general anesthesia.^24^ Although the patient did not provide specific information about the duration of symptoms, thanks to the information given we supposed that the incident had not happened during the induction or at the emergence of the anesthesia. The psychologist's interview with the anesthesists confirmed our hypothesis, as he described his error in the administration of inhaled anesthetics during the maintenance phase. Because the monitoring of inhaled anesthetic was not applied, the sudden arousal from the anesthesia status was not promptly detect, despite the suggestive presence of other clinical and instrumental data. Moreover, the anesthesia was performed without any device for DOA monitoring. Thus, in this case, the combination of standard clinical and instrumental monitoring (included inhaled anesthetic monitoring) with DOA monitoring most likely could have prevented the unpleasant complication.

The psychological consequences of a GAAWR episode vary. Although some patients do not show any sign of mental discomfort, others develop psychological problems or severe and persistent psychiatric sequelae.^25^ These consequences may include anxiety, insomnia, flashbacks, or hyper-arousal, which can trigger a post-traumatic stress disorders syndrome (PTSD).^26^ According to Aceto and colleagues the prevalence of PTSD ranged from 0 to 71%.^27^ Thus, there are no accurate data in the literature. The reason of this lack is the difficulty to follow all patients according to a specific psychological assessment. Indeed, the patients may not remember our intraoperative experience until days, weeks or several months after anesthesia. In a cancer hospital, patients can benefit from a psychological support, even after surgery and during follow-up. As a consequence, the possibility of including specific questions in the psychological tools commonly used by psycho-oncologists could be 1 more weapon for detecting psychological or psychiatric consequences after surgery, which might remain unrecognized. A significant data of our analysis is the poor of psychological consequences and more serious psychiatric sequelae, such as PTSD, related to GAAWR. Although the first case of anesthesia awareness (case 1) did not present sequelae, case 2 manifested intraoperative distress, consisting in sense of suffocation and paralysis, and most likely these experiences caused a sense of discomfort in the immediate postoperative period. Mental health interventions are the “gold” standard of care for managing mental health symptoms that may be brought on by GAAWR.^28^ Thanks to a professional intervention these symptoms were quickly resolved and the patient had no psychological consequences.

Another significant data that emerges from our analysis concerns the limited use of DOA monitoring. Indeed, DOA devices were used only in 0.65% of patients. This data is still lower than that detected in UK where specific DOA monitors are used in 2.8% of general anesthesia.^9^ In both case reports, after premedication with midazolam and fentanyl the anesthesia management was performed with propofol at the induction followed by sevoflurane and fentanyl for maintenance; however, anesthesists did not use neuromuscular block and DOA monitoring. A practical observation is that in case 2 the combination of inhaled gas and DOA monitoring could have avoided the complication.

### Limitations of the Study

Considering that our study is a retrospective analysis, the limitation in examining only spontaneous reports of GAAWR is a paramount obstacle to an exhaustive collection of cases. This is the subject of a great debate between the authors of the NAP5 study and the major scholars on the topic of the anesthesia awareness, like Avidan and Sleigh.^29^ Indeed, many patients may not choose to discuss their experience unless they are asked directly about it, even in >1 occasion.^30^ Another limit is the lack of a specific registry for self-reporting of GAAWR, like that of Kent and colleagues.^31^ However, all the patients treated at the Istituto Nazionale Tumori of Naples usually receive an efficient psychological assistance (psycho-oncology), during the medical treatments and the whole care setting. Consequently, any discomfort perceived in the perioperative time can still come to light throughout the various conversations between the cancer patient and the psychologist, even if not requested specifically through structured interviews, like Brice interview.^32^ Yet, because the onset time of psychological symptoms vary widely from 7 to 243 days after surgery, and the median duration of psychological symptoms ranges from 4.4 to 5.6 years,^7,33^ a psychological assessment obtained through various interviews over time may be particularly effective to unmask every condition of psychological distress.

## CONCLUSIONS

Despite the limitations, our investigation suggests that the incidence of anesthesia awareness is very low, also in a specific cohort a patients, for example the cancer patients, as well as even when DOA monitoring is rarely used. This evidence is also observed in emergency patients and in procedures performed under sedation. Moreover, because 1 of the 2 cases refers to an error in drug administration of inhaled anesthetics it could be easily avoided.

A valid postoperative psychological assessment, often routinely applied in a cancer hospital, could fill in for the specific tools for direct awareness detection, such as structured interviews, the lack of which, together with the retrospective analysis, represents a limit of the study. Moreover, this observation could suggest the usefulness to include specific questions in the psychological tools commonly used by psycho-oncologists, with the aim to detect psychiatric or psychological awareness consequences which might remain unrecognized, compounding the psychological status of cancer patients. The next step of our investigation is to develop a specific tool (eg a questionnaire) with the purpose of allowing both the psycho-oncological assessment and the quick detecting of anesthesia awareness and its consequences in cancer patients.

### Key Messages

In a retrospective analysis on a large cohort of cancer patients in a single center the incidence of GAAWR is very low, despite the rare use of specific DOA monitors.

A strong postoperative psychological assessment can minimize the lack of data due to the nonapplication of specific tools for anesthesia awareness detection. This observation could suggest the usefulness of inserting specific questions within the psychological tools commonly used by psycho-oncologists.

This retrospective study shows that the incidence of GAAWR is low also during emergency surgery and in procedures performed under sedation.
